# 1-Butyl-3-methyl­imidazolium tri­bromido­(tri­phenyl­phosphane-κ*P*)nickelate(II) butan-1-ol hemisolvate

**DOI:** 10.1107/S241431462100818X

**Published:** 2021-08-13

**Authors:** Tim Peppel, Martin Köckerling

**Affiliations:** a Leibniz-Institut für Katalyse e.V. (LIKAT), Heterogene Photokatalyse, Albert-Einstein-Str. 29a, D-18059 Rostock, Germany; b Universität Rostock, Institut für Chemie, Anorganische Festkörperchemie, Albert-Einstein-Str. 3a, D-18059 Rostock, Germany; Vienna University of Technology, Austria

**Keywords:** crystal structure, nickel, coordination compound, ionic liquid

## Abstract

The solvated title salt, (C_8_H_15_N_2_)[NiBr_3_(P(C_6_H_5_)_3_)]·0.5C_4_H_10_O, was obtained in the form of single crystals directly from the reaction mixture. The mol­ecular structure consists of separated 1-butyl-3-methyl­imidazolium cations, tri­bromido­(tri­phenyl­phosphane)nickelate(II) anions and half a solvent mol­ecule of 1-butanol, all connected *via* multiple hydrogen contacts to form a three-dimensional network. The co-crystallized 1-butanol mol­ecule is disordered and adopts two orientations. The central C—C bonds of both orientations are located on an inversion centre (Wyckoff site 2*b* of space group *P*2_1_/*n*). Thereby, each orientation has again two orientations with the OH group being located either on one or the other side of the C_4_ alkyl chain. The dried solvent-free compound exhibits a relatively low melting point (m.p. = 412 K).

## Abstract 2

The title compound consists of 1-butyl-3-methyl­imidazolium cations, tri­bromido­(tri­phenyl­phophine)nickelate(II) anions and co-crystallized 1-butanol solvent mol­ecules.

## Graphical abstract

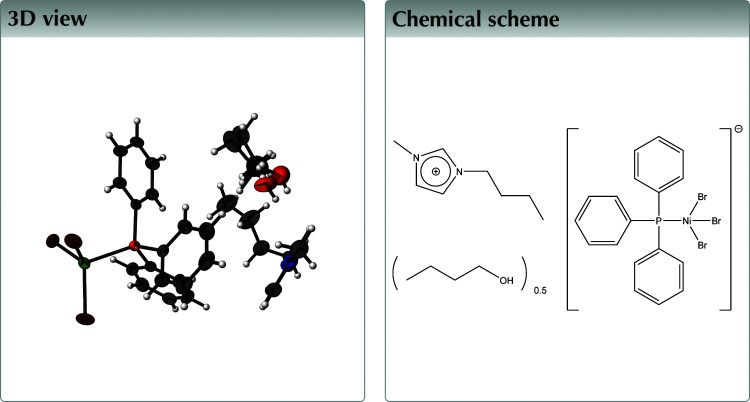

## Structure description

Nickel(II) complexes exhibiting pseudo-tetra­hedral symmetry [Ni*X*_3_ *L*]^−^ [*X*: halide, *L*: neutral ligand, *e.g*. P(C_6_H_5_)_3_] have been thoroughly characterized for more than 50 years by means of magnetic investigations, UV–vis and far-infrared spectroscopy, NMR and EPR as well as Mössbauer spectroscopy (Figgis *et al.*, 1966 ▸; Bradbury *et al.*, 1967 ▸; Fischer & Horrocks, 1968 ▸; Erich *et al.*, 1969 ▸; Gerloch *et al.*, 1981 ▸; Desrochers *et al.*, 2006 ▸). In addition, complexes bearing the [NiBr_3_P(C_6_H_5_)_3_]^−^ anion have been investigated as suitable precatalysts for the generation of Ni–NHC complexes (NHC: *N*-heterocyclic carbene) in catalytic processes, *e.g.* selective cross-coupling reactions (Xu *et al.*, 2013 ▸; Poulten *et al.*, 2014 ▸; Zhang *et al.*, 2015 ▸). Single-crystal structure determinations of complexes with the general formula (*cation*)[NiBr_3_P(C_6_H_5_)_3_] are known for the following cations: [As(C_6_H_5_)_4_]^+^ (Hanton & Raithby, 1980 ▸), (DiPIm)^+^ (DiPIm: 1,3-diiso­propyl­imidazolium; Xu *et al.*, 2013 ▸), (DiPPhIm)^+^ (DiPPhIm: 1,3-bis­(2,6-diiso­propyl­phen­yl)imidazolium; Xu *et al.*, 2013 ▸), and (EMIm)^+^ (EMIm: 1-ethyl-3-methyl­imidazolium; Peppel *et al.*, 2013 ▸). We report here the synthesis and crystal structure of (BMIm)^+^[NiBr_3_(P(C_6_H_5_)_3_)]^−^·0.5(C_4_H_10_O) (BMIm^+^ is 1-butyl-3-methyl­imidazolium).

**Figure 1:**
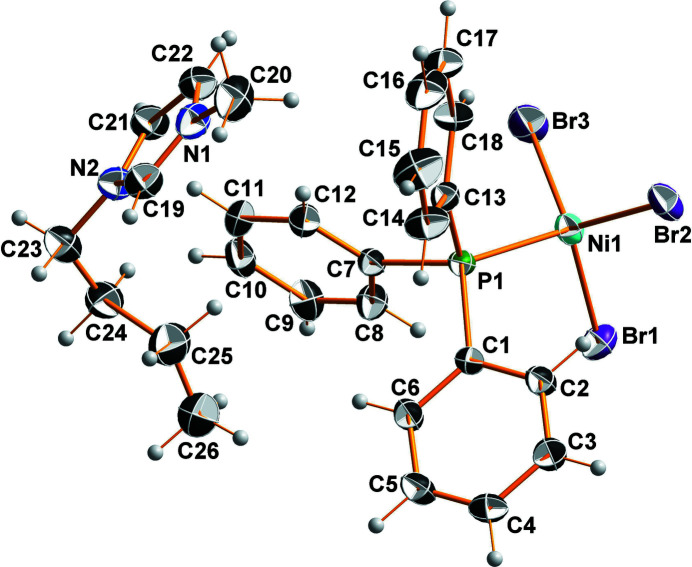
The structure of the mol­ecular cation–anion pair in (BMIm)[NiBr_3_(P(C_6_H_5_)_3_)]·0.5(C_4_H_10_O). Displacement ellipsoids are drawn at the 50% probability level; the disordered co-crystallized 1-butanol mol­ecule has been omitted for clarity.

**Figure 2:**
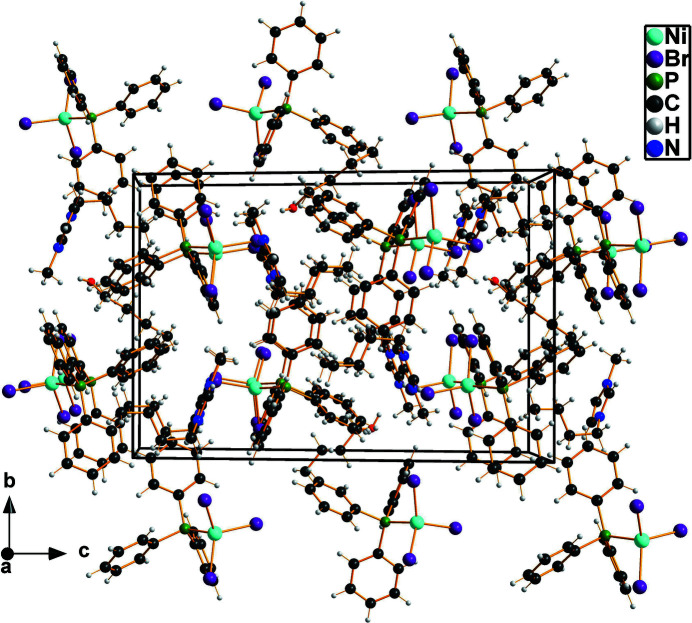
A view of the unit-cell contents of the title compound in a projection down the *a* axis.

The asymmetric unit of the title compound consists of one 1-butyl-3-methyl­imidazolium cation and one tri­bromido­(tri­phenyl­phosphane)nickelate(II) anion (Fig. 1 ▸). An additional highly distorted half mol­ecule of 1-butanol is incorporated in the crystal structure. Together with weak C—H⋯Br contacts involving the anion, the OH function of the solvent mol­ecule forms hydrogen bonds to the N atom of the cation, building up an extended three-dimensional hydrogen-bonded network (Table 1 ▸). The co-crystallized 1-butanol mol­ecule adopts two orientations. The central C—C bonds of both orientations are located on the inversion centre whereby each orientation has again two orientations with the OH group being located either on one or the other side of the C_4_ alkyl chain. All bond lengths and angles within the cation as well as the complex anion are in the expected ranges (Peppel *et al.*, 2013 ▸). The coordination environment around the Ni^II^ atom is pseudo-tetra­hedral with Br—Ni—Br angles ranging from 109.95 (2) to 117.94 (2)°, and Br—Ni—P angles ranging from 102.76 (2) to 106.26 (2)°. The packing of the mol­ecular entities is depicted in Fig. 2 ▸.

## Synthesis and crystallization

The title compound was obtained as blue crystals in multi-gram scale from 1-butyl-3-methyl­imidazolium bromide, tri­phenyl­phosphane and anhydrous nickel(II) bromide in boiling 1-butanol (Peppel *et al.*, 2013 ▸). 1-Butyl-3-methyl­imidazolium bromide (1.0 g, 4.6 mmol) and tri­phenyl­phosphane (1.2 g, 4.6 mmol) were dissolved in 20 ml of 1-butanol in a Schlenk tube. This solution was added in one portion to a vigorously stirred, nearly boiling suspension of NiBr_2_ (1.0 g, 4.6 mmol) in 30 ml of 1-butanol. The resulting green precipitate was completely dissolved by heating up the suspension to the boiling point of the solvent. The hot solution was cooled down to 277 K overnight in a refrigerator. The resulting blue crystals were filtered off, washed thoroughly with diethyl ether and dried in vacuum at ambient conditions (2.5 g, 78%).

Analytic data for C_26_H_30_Br_3_N_2_NiP: m.p. 412 K, elemental analysis % (calc.): C 42.61 (44.62); H 4.28 (4.32); N 4.18 (4.00).

## Refinement

Crystal data, data collection and structure refinement details are summarized in Table 2 ▸. Several low-angle reflections were omitted in the structure refinement because their intensities were affected by the beam stop. The centre of the co-crystallized 1-butanol mol­ecule is located on the Wyckoff site 2*b* of space group *P*2_1_/*n*. It is disordered with two different orientations, which were refined using a split arrangement with the sum of occupational factors being fixed to full occupation. This results in a total of half a mol­ecule per formula unit. The H atoms of the disordered 1-butanol mol­ecule including that attached to the O atom were calculated at idealized positions and refined using riding models.

## Supplementary Material

Crystal structure: contains datablock(s) I. DOI: 10.1107/S241431462100818X/wm4149sup1.cif

Structure factors: contains datablock(s) I. DOI: 10.1107/S241431462100818X/wm4149Isup2.hkl

CCDC reference: 2102393

Additional supporting information:  crystallographic information; 3D view; checkCIF report

## Figures and Tables

**Table 1:** Hydrogen-bond geometry (Å, °)

*D*—H⋯*A*	*D*—H	H⋯*A*	*D*⋯*A*	*D*—H⋯*A*
O1*B*—H1*BA*⋯N1	0.85	2.68	3.36 (1)	139
C19—H19*A*⋯O1*A*	0.95	2.59	3.17 (1)	120
C2—H2*A*⋯Br2	0.95	2.95	3.817 (2)	153
C23—H23*A*⋯Br3^i^	0.99	2.84	3.718 (3)	148
C20—H20*A*⋯Br1^ii^	0.95	3.02	3.906 (3)	156
C8—H8*A*⋯Br1	0.95	3.08	3.921 (2)	149
C23—H23*B*⋯Br2^iii^	0.99	3.13	3.711 (3)	119

Symmetry codes: (i) 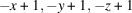 ; (ii) 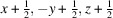 ; (iii) 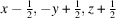 .

**Table 2:** Experimental details

Crystal data	
Chemical formula	(C_8_H_15_N_2_)[NiBr_3_(C_18_H_15_P)]·0.5C_4_H_10_O
*M*_r_	736.99
Crystal system, space group	Monoclinic, *P*2_1_/*n*
Temperature (K)	173
*a*, *b*, *c* (Å)	9.9571 (4), 14.4731 (6), 21.4730 (9)
β (°)	102.620 (2)
*V* (Å^3^)	3019.7 (2)
*Z*	4
Radiation type	Mo *K*α
μ (mm^−1^)	4.69
Crystal size (mm)	0.40 × 0.35 × 0.20

Data collection	
Diffractometer	Bruker APEX CCD
Absorption correction	Multi-scan (*SADABS*; Krause *et al.*, 2015 ▸)
No. of measured, independent and observed [*I* > 2σ(*I*)] reflections	44088, 6832, 5812
*R*_int_	0.027
(sin θ/λ)_max_ (Å^−1^)	0.648

Refinement	
*R*[*F* ^2^ > 2σ(*F* ^2^)], *wR*(*F* ^2^), *S*	0.028, 0.068, 1.04
No. of reflections	6832
No. of parameters	352
H-atom treatment	H-atom parameters constrained
Δρ_max_, Δρ_min_ (e Å^−3^)	1.27, −0.72

Computer programs: *APEX2* (Bruker, 2007 ▸), *SAINT* (Bruker, 2007 ▸), *SHELXT* (Sheldrick, 2015*a* ▸), *SHELXL* (Sheldrick, 2015*b* ▸), *DIAMOND* (Brandenburg & Putz, 2019 ▸) and *publCIF* (Westrip, 2010 ▸).

## full crystallographic data

### Crystal data

**Table:**

(C_8_H_15_N_2_)[NiBr_3_(C_18_H_15_P)]·0.5C_4_H_10_O	*F*(000) = 1476
*M**_r_* = 736.99	*D*_x_ = 1.621 Mg m^−^^3^
Monoclinic, *P*2_1_/*n*	Mo *K*α radiation, λ = 0.71073 Å
*a* = 9.9571 (4) Å	Cell parameters from 9717 reflections
*b* = 14.4731 (6) Å	θ = 3.4–27.3°
*c* = 21.4730 (9) Å	µ = 4.69 mm^−^^1^
β = 102.620 (2)°	*T* = 173 K
*V* = 3019.7 (2) Å^3^	Block, blue
*Z* = 4	0.40 × 0.35 × 0.20 mm

### Data collection

**Table:**

Bruker APEX CCD diffractometer	5812 reflections with *I* > 2σ(*I*)
Radiation source: sealed tube	*R*_int_ = 0.027
φ and ω scans	θ_max_ = 27.4°, θ_min_ = 3.2°
Absorption correction: multi-scan (*SADABS*; Krause *et al.*, 2015)	*h* = −12→11
	*k* = −18→18
44088 measured reflections	*l* = −27→27
6832 independent reflections	

### Refinement

**Table:**

Refinement on *F*^2^	Primary atom site location: structure-invariant direct methods
Least-squares matrix: full	Secondary atom site location: difference Fourier map
*R*[*F*^2^ > 2σ(*F*^2^)] = 0.028	Hydrogen site location: mixed
*wR*(*F*^2^) = 0.068	H-atom parameters constrained
*S* = 1.04	*w* = 1/[σ^2^(*F*_o_^2^) + (0.0282*P*)^2^ + 3.0233*P*] where *P* = (*F*_o_^2^ + 2*F*_c_^2^)/3
6832 reflections	(Δ/σ)_max_ = 0.001
352 parameters	Δρ_max_ = 1.27 e Å^−^^3^
0 restraints	Δρ_min_ = −0.72 e Å^−^^3^

### Special details

**Table:**

Geometry. All esds (except the esd in the dihedral angle between two l.s. planes) are estimated using the full covariance matrix. The cell esds are taken into account individually in the estimation of esds in distances, angles and torsion angles; correlations between esds in cell parameters are only used when they are defined by crystal symmetry. An approximate (isotropic) treatment of cell esds is used for estimating esds involving l.s. planes.

### Fractional atomic coordinates and isotropic or equivalent isotropic displacement parameters (Å^2^)

**Table:**

	*x*	*y*	*z*	*U*_iso_*/*U*_eq_	Occ. (<1)
Ni1	0.51042 (3)	0.23911 (2)	0.30017 (2)	0.02846 (8)	
Br1	0.35880 (3)	0.26583 (2)	0.19977 (2)	0.03852 (7)	
Br2	0.59646 (3)	0.08690 (2)	0.31181 (2)	0.04115 (7)	
Br3	0.66974 (3)	0.36192 (2)	0.32458 (2)	0.04823 (8)	
P1	0.37606 (5)	0.24590 (4)	0.37386 (3)	0.0227 (1)	
C1	0.2154 (2)	0.1831 (2)	0.3494 (1)	0.0240 (4)	
C2	0.2199 (2)	0.0952 (2)	0.3241 (1)	0.0306 (5)	
H2A	0.3054	0.0700	0.3196	0.037*	
C3	0.1000 (2)	0.0439 (2)	0.3053 (1)	0.0347 (5)	
H3A	0.1035	−0.0165	0.2884	0.042*	
C4	−0.0248 (2)	0.0811 (2)	0.3113 (1)	0.0345 (5)	
H4A	−0.1070	0.0464	0.2979	0.041*	
C5	−0.0299 (2)	0.1678 (2)	0.3364 (1)	0.0377 (6)	
H5A	−0.1157	0.1927	0.3407	0.045*	
C6	0.0896 (2)	0.2193 (2)	0.3556 (1)	0.0328 (5)	
H6A	0.0855	0.2793	0.3730	0.039*	
C7	0.3318 (2)	0.3640 (2)	0.3896 (1)	0.0259 (4)	
C8	0.2805 (3)	0.4208 (2)	0.3374 (1)	0.0346 (5)	
H8A	0.2655	0.3961	0.2954	0.042*	
C9	0.2512 (3)	0.5130 (2)	0.3460 (1)	0.0399 (6)	
H9A	0.2144	0.5508	0.3102	0.048*	
C10	0.2757 (3)	0.5495 (2)	0.4069 (1)	0.0388 (6)	
H10A	0.2569	0.6128	0.4130	0.047*	
C11	0.3272 (3)	0.4941 (2)	0.4586 (1)	0.0409 (6)	
H11A	0.3443	0.5198	0.5003	0.049*	
C12	0.3545 (3)	0.4013 (2)	0.4508 (1)	0.0351 (5)	
H12A	0.3884	0.3634	0.4869	0.042*	
C13	0.4593 (2)	0.1969 (2)	0.4503 (1)	0.0276 (5)	
C14	0.3923 (3)	0.1379 (2)	0.4843 (1)	0.0438 (6)	
H14A	0.2977	0.1240	0.4687	0.053*	
C15	0.4633 (4)	0.0992 (2)	0.5413 (1)	0.0566 (8)	
H15A	0.4175	0.0581	0.5643	0.068*	
C16	0.5996 (3)	0.1203 (2)	0.5645 (1)	0.0504 (7)	
H16A	0.6479	0.0939	0.6035	0.060*	
C17	0.6659 (3)	0.1792 (2)	0.5315 (1)	0.0465 (7)	
H17A	0.7597	0.1944	0.5482	0.056*	
C18	0.5976 (3)	0.2172 (2)	0.4739 (1)	0.0382 (6)	
H18A	0.6451	0.2568	0.4508	0.046*	
N1	0.3974 (2)	0.2444 (2)	0.6874 (1)	0.0386 (5)	
C19	0.2707 (3)	0.2755 (2)	0.6827 (1)	0.0404 (6)	
H19A	0.1975	0.2423	0.6942	0.048*	
N2	0.2626 (2)	0.3606 (1)	0.6592 (1)	0.0364 (5)	
C20	0.4739 (3)	0.3115 (2)	0.6664 (1)	0.0416 (6)	
H20A	0.5682	0.3077	0.6646	0.050*	
C21	0.3894 (3)	0.3840 (2)	0.6486 (1)	0.0396 (6)	
H21A	0.4131	0.4409	0.6317	0.048*	
C22	0.4472 (4)	0.1537 (2)	0.7127 (2)	0.0542 (8)	
H22A	0.3718	0.1196	0.7249	0.065*	
H22B	0.5224	0.1618	0.7502	0.065*	
H22C	0.4806	0.1190	0.6799	0.065*	
C23	0.1370 (3)	0.4170 (2)	0.6441 (1)	0.0425 (6)	
H23A	0.1577	0.4798	0.6620	0.051*	
H23B	0.0665	0.3893	0.6645	0.051*	
C24	0.0810 (3)	0.4240 (2)	0.5740 (1)	0.0485 (7)	
H24A	0.1509	0.4524	0.5535	0.058*	
H24B	−0.0012	0.4644	0.5658	0.058*	
C25	0.0420 (4)	0.3292 (3)	0.5448 (2)	0.073 (1)	
H25A	0.1277	0.2961	0.5422	0.088*	
H25B	−0.0027	0.2938	0.5741	0.088*	
C26	−0.0481 (4)	0.3284 (3)	0.4823 (2)	0.080 (1)	
H26A	−0.0656	0.2644	0.4679	0.096*	
H26B	−0.0046	0.3619	0.4523	0.096*	
H26C	−0.1353	0.3583	0.4843	0.096*	
O1A	0.055 (1)	0.1262 (8)	0.6149 (6)	0.083 (3)	0.25
H1AA	0.1311	0.1364	0.6405	0.125*	0.25
C27A	0.093 (3)	0.072 (2)	0.578 (2)	0.10 (1)	0.25
H27A	0.1412	0.1113	0.5539	0.121*	0.25
H27B	0.1560	0.0260	0.5980	0.121*	0.25
C27C	0.093 (3)	0.072 (2)	0.578 (2)	0.10 (1)	0.25
H27E	0.0615	0.1179	0.5964	0.121*	0.25
H27F	0.1616	0.0354	0.5927	0.121*	0.25
H27G	0.1694	0.1257	0.5533	0.121*	0.25
C28A	−0.0246 (9)	0.0276 (5)	0.5250 (4)	0.061 (2)	0.5
H28A	−0.0800	0.0795	0.5028	0.073*	0.5
H28B	−0.0879	−0.0121	0.5428	0.073*	0.5
O1B	0.192 (1)	0.0781 (8)	0.6091 (6)	0.069 (3)	0.25
H1BA	0.1987	0.1296	0.6285	0.103*	0.25
C27B	0.089 (2)	0.077 (2)	0.568 (1)	0.056 (4)	0.25
H27C	0.0791	0.1259	0.5426	0.067*	0.25
H27D	0.0119	0.0687	0.5882	0.067*	0.25
C27D	0.089 (2)	0.077 (2)	0.568 (1)	0.056 (4)	0.25
H27H	0.1726	0.0648	0.6010	0.067*	0.25
H27I	0.0881	0.1287	0.5476	0.067*	0.25
H27J	0.0133	0.0753	0.5938	0.067*	0.25
C28B	0.0679 (9)	−0.0022 (5)	0.5252 (4)	0.069 (2)	0.5
H28C	0.0687	−0.0595	0.5478	0.083*	0.5
H28D	0.1424	−0.0040	0.5034	0.083*	0.5

### Atomic displacement parameters (Å^2^)

**Table:**

	*U*^11^	*U*^22^	*U*^33^	*U*^12^	*U*^13^	*U*^23^
Ni1	0.0268 (2)	0.0286 (2)	0.0320 (2)	−0.0005 (1)	0.0109 (1)	0.0003 (1)
Br1	0.0476 (2)	0.0360 (1)	0.0296 (1)	−0.0026 (1)	0.0032 (1)	−0.0005 (1)
Br2	0.0331 (1)	0.0308 (1)	0.0628 (2)	0.0057 (1)	0.0177 (1)	0.0017 (1)
Br3	0.0392 (2)	0.0335 (1)	0.0747 (2)	−0.0103 (1)	0.0182 (1)	−0.0062 (1)
P1	0.0204 (3)	0.0233 (3)	0.0240 (3)	−0.0001 (2)	0.0040 (2)	−0.0001 (2)
C1	0.022 (1)	0.027 (1)	0.023 (1)	−0.0009 (8)	0.0045 (8)	0.0028 (8)
C2	0.024 (1)	0.029 (1)	0.039 (1)	0.0000 (9)	0.0083 (9)	−0.001 (1)
C3	0.033 (1)	0.029 (1)	0.043 (1)	−0.006 (1)	0.010 (1)	−0.004 (1)
C4	0.027 (1)	0.038 (1)	0.039 (1)	−0.009 (1)	0.006 (1)	0.003 (1)
C5	0.025 (1)	0.041 (1)	0.049 (2)	0.001 (1)	0.011 (1)	0.001 (1)
C6	0.028 (1)	0.031 (1)	0.041 (1)	0.0009 (9)	0.012 (1)	−0.004 (1)
C7	0.022 (1)	0.026 (1)	0.031 (1)	−0.0003 (8)	0.0071 (9)	−0.0020 (9)
C8	0.041 (1)	0.030 (1)	0.033 (1)	0.002 (1)	0.008 (1)	−0.001 (1)
C9	0.047 (2)	0.030 (1)	0.045 (1)	0.006 (1)	0.015 (1)	0.007 (1)
C10	0.038 (1)	0.026 (1)	0.058 (2)	0.001 (1)	0.021 (1)	−0.004 (1)
C11	0.042 (1)	0.040 (1)	0.040 (1)	0.002 (1)	0.008 (1)	−0.015 (1)
C12	0.036 (1)	0.036 (1)	0.032 (1)	0.005 (1)	0.005 (1)	−0.004 (1)
C13	0.030 (1)	0.027 (1)	0.024 (1)	0.0025 (9)	0.0030 (9)	−0.0013 (9)
C14	0.042 (2)	0.054 (2)	0.033 (1)	−0.009 (1)	0.003 (1)	0.009 (1)
C15	0.072 (2)	0.061 (2)	0.034 (1)	−0.010 (2)	0.004 (1)	0.015 (1)
C16	0.063 (2)	0.051 (2)	0.029 (1)	0.011 (1)	−0.008 (1)	0.004 (1)
C17	0.039 (2)	0.055 (2)	0.037 (1)	0.008 (1)	−0.009 (1)	−0.004 (1)
C18	0.032 (1)	0.043 (1)	0.036 (1)	0.000 (1)	−0.001 (1)	0.003 (1)
N1	0.045 (1)	0.032 (1)	0.038 (1)	0.0001 (9)	0.0055 (9)	−0.0016 (9)
C19	0.042 (2)	0.038 (1)	0.042 (1)	−0.007 (1)	0.010 (1)	0.002 (1)
N2	0.034 (1)	0.035 (1)	0.040 (1)	−0.0032 (9)	0.0071 (9)	0.0012 (9)
C20	0.036 (1)	0.042 (1)	0.046 (2)	−0.005 (1)	0.007 (1)	−0.004 (1)
C21	0.038 (1)	0.035 (1)	0.047 (2)	−0.007 (1)	0.010 (1)	0.001 (1)
C22	0.070 (2)	0.035 (1)	0.056 (2)	0.009 (1)	0.009 (2)	0.002 (1)
C23	0.035 (1)	0.038 (1)	0.056 (2)	0.001 (1)	0.014 (1)	0.004 (1)
C24	0.044 (2)	0.044 (2)	0.054 (2)	0.009 (1)	0.001 (1)	−0.003 (1)
C25	0.066 (2)	0.062 (2)	0.080 (2)	0.020 (2)	−0.010 (2)	−0.023 (2)
C26	0.062 (2)	0.080 (3)	0.093 (3)	0.006 (2)	0.006 (2)	−0.036 (2)
O1A	0.095 (9)	0.078 (7)	0.083 (8)	−0.012 (6)	0.033 (6)	−0.021 (7)
C27A	0.17 (2)	0.054 (9)	0.09 (2)	−0.03 (1)	0.05 (2)	0.01 (1)
C27C	0.17 (2)	0.054 (9)	0.09 (2)	−0.03 (1)	0.05 (2)	0.01 (1)
C28A	0.059 (5)	0.062 (4)	0.064 (5)	0.011 (4)	0.019 (4)	0.009 (4)
O1B	0.057 (6)	0.058 (6)	0.080 (8)	−0.012 (5)	−0.011 (6)	−0.003 (5)
C27B	0.079 (9)	0.047 (8)	0.047 (5)	0.012 (7)	0.027 (5)	0.008 (5)
C27D	0.079 (9)	0.047 (8)	0.047 (5)	0.012 (7)	0.027 (5)	0.008 (5)
C28B	0.091 (6)	0.046 (4)	0.084 (7)	0.014 (4)	0.048 (5)	0.013 (4)

### Geometric parameters (Å, º)

**Table:**

Ni1—P1	2.2865 (6)	C19—H19A	0.9500
Ni1—Br2	2.3570 (4)	N2—C21	1.373 (3)
Ni1—Br3	2.3638 (4)	N2—C23	1.469 (3)
Ni1—Br1	2.3779 (4)	C20—C21	1.347 (4)
P1—C13	1.814 (2)	C20—H20A	0.9500
P1—C1	1.815 (2)	C21—H21A	0.9500
P1—C7	1.815 (2)	C22—H22A	0.9800
C1—C2	1.389 (3)	C22—H22B	0.9800
C1—C6	1.390 (3)	C22—H22C	0.9800
C2—C3	1.389 (3)	C23—C24	1.490 (4)
C2—H2A	0.9500	C23—H23A	0.9900
C3—C4	1.385 (3)	C23—H23B	0.9900
C3—H3A	0.9500	C24—C25	1.524 (4)
C4—C5	1.372 (4)	C24—H24A	0.9900
C4—H4A	0.9500	C24—H24B	0.9900
C5—C6	1.388 (3)	C25—C26	1.441 (5)
C5—H5A	0.9500	C25—H25A	0.9900
C6—H6A	0.9500	C25—H25B	0.9900
C7—C12	1.392 (3)	C26—H26A	0.9800
C7—C8	1.394 (3)	C26—H26B	0.9800
C8—C9	1.387 (3)	C26—H26C	0.9800
C8—H8A	0.9500	O1A—C27A	1.23 (3)
C9—C10	1.381 (4)	O1A—H1AA	0.8500
C9—H9A	0.9500	O1A—H27E	0.4348
C10—C11	1.376 (4)	C27A—C28A	1.58 (4)
C10—H10A	0.9500	C27A—H27A	0.9600
C11—C12	1.388 (3)	C27A—H27B	0.9601
C11—H11A	0.9500	C27A—H27E	0.8595
C12—H12A	0.9500	C27A—H27F	0.8765
C13—C14	1.386 (3)	C27A—H27G	1.2768
C13—C18	1.392 (3)	C28A—H28A	0.9900
C14—C15	1.390 (4)	C28A—H28B	0.9900
C14—H14A	0.9500	O1B—C27B	1.20 (3)
C15—C16	1.375 (4)	O1B—H1BA	0.8499
C15—H15A	0.9500	O1B—H27H	0.2980
C16—C17	1.367 (4)	C27B—C28B	1.46 (3)
C16—H16A	0.9500	C27B—H27C	0.8813
C17—C18	1.388 (4)	C27B—H27D	0.9708
C17—H17A	0.9500	C27B—H27H	0.9844
C18—H18A	0.9500	C27B—H27I	0.8620
N1—C19	1.322 (4)	C27B—H27J	1.0301
N1—C20	1.371 (3)	C28B—H28C	0.9599
N1—C22	1.465 (3)	C28B—H28D	0.9601
C19—N2	1.327 (3)		
			
P1—Ni1—Br2	102.76 (2)	H22A—C22—H22B	109.5
P1—Ni1—Br3	106.26 (2)	N1—C22—H22C	109.5
Br2—Ni1—Br3	117.94 (2)	H22A—C22—H22C	109.5
P1—Ni1—Br1	105.60 (2)	H22B—C22—H22C	109.5
Br2—Ni1—Br1	113.09 (2)	N2—C23—C24	112.0 (2)
Br3—Ni1—Br1	109.95 (2)	N2—C23—H23A	109.2
C13—P1—C1	105.4 (1)	C24—C23—H23A	109.2
C13—P1—C7	106.4 (1)	N2—C23—H23B	109.2
C1—P1—C7	106.9 (1)	C24—C23—H23B	109.2
C13—P1—Ni1	112.87 (8)	H23A—C23—H23B	107.9
C1—P1—Ni1	112.98 (7)	C23—C24—C25	111.1 (3)
C7—P1—Ni1	111.83 (7)	C23—C24—H24A	109.4
C2—C1—C6	119.3 (2)	C25—C24—H24A	109.4
C2—C1—P1	118.0 (2)	C23—C24—H24B	109.4
C6—C1—P1	122.7 (2)	C25—C24—H24B	109.4
C3—C2—C1	120.3 (2)	H24A—C24—H24B	108.0
C3—C2—H2A	119.9	C26—C25—C24	116.2 (3)
C1—C2—H2A	119.9	C26—C25—H25A	108.2
C4—C3—C2	119.8 (2)	C24—C25—H25A	108.2
C4—C3—H3A	120.1	C26—C25—H25B	108.2
C2—C3—H3A	120.1	C24—C25—H25B	108.2
C5—C4—C3	120.2 (2)	H25A—C25—H25B	107.4
C5—C4—H4A	119.9	C25—C26—H26A	109.5
C3—C4—H4A	119.9	C25—C26—H26B	109.5
C4—C5—C6	120.3 (2)	H26A—C26—H26B	109.5
C4—C5—H5A	119.8	C25—C26—H26C	109.5
C6—C5—H5A	119.8	H26A—C26—H26C	109.5
C5—C6—C1	120.1 (2)	H26B—C26—H26C	109.5
C5—C6—H6A	120.0	C27A—O1A—H1AA	99.8
C1—C6—H6A	120.0	C27A—O1A—H27E	25.8
C12—C7—C8	119.1 (2)	H1AA—O1A—H27E	109.9
C12—C7—P1	123.0 (2)	O1A—C27A—C28A	116 (2)
C8—C7—P1	117.8 (2)	O1A—C27A—H27A	103.9
C9—C8—C7	120.7 (2)	C28A—C27A—H27A	103.7
C9—C8—H8A	119.6	O1A—C27A—H27B	114.6
C7—C8—H8A	119.6	C28A—C27A—H27B	110.2
C10—C9—C8	119.7 (2)	H27A—C27A—H27B	107.0
C10—C9—H9A	120.1	O1A—C27A—H27E	12.7
C8—C9—H9A	120.1	C28A—C27A—H27E	111.3
C11—C10—C9	119.9 (2)	H27A—C27A—H27E	94.5
C11—C10—H10A	120.0	H27B—C27A—H27E	126.4
C9—C10—H10A	120.0	O1A—C27A—H27F	118.6
C10—C11—C12	120.9 (2)	C28A—C27A—H27F	114.1
C10—C11—H11A	119.5	H27A—C27A—H27F	96.1
C12—C11—H11A	119.5	H27B—C27A—H27F	10.9
C11—C12—C7	119.6 (2)	H27E—C27A—H27F	129.1
C11—C12—H12A	120.2	O1A—C27A—H27G	100.5
C7—C12—H12A	120.2	C28A—C27A—H27G	111.6
C14—C13—C18	119.3 (2)	H27A—C27A—H27G	8.0
C14—C13—P1	122.8 (2)	H27B—C27A—H27G	102.4
C18—C13—P1	117.9 (2)	H27E—C27A—H27G	92.3
C13—C14—C15	120.1 (3)	H27F—C27A—H27G	91.5
C13—C14—H14A	119.9	C27A—C28A—H28A	106.3
C15—C14—H14A	119.9	C27A—C28A—H28B	113.3
C16—C15—C14	120.1 (3)	H28A—C28A—H28B	107.0
C16—C15—H15A	120.0	C27B—O1B—H1BA	109.7
C14—C15—H15A	120.0	C27B—O1B—H27H	39.7
C17—C16—C15	120.1 (2)	H1BA—O1B—H27H	145.9
C17—C16—H16A	119.9	O1B—C27B—C28B	117 (1)
C15—C16—H16A	119.9	O1B—C27B—H27C	114.4
C16—C17—C18	120.7 (3)	C28B—C27B—H27C	105.0
C16—C17—H17A	119.7	O1B—C27B—H27D	107.6
C18—C17—H17A	119.7	C28B—C27B—H27D	99.2
C17—C18—C13	119.7 (3)	H27C—C27B—H27D	112.7
C17—C18—H18A	120.2	O1B—C27B—H27H	11.2
C13—C18—H18A	120.2	C28B—C27B—H27H	107.0
C19—N1—C20	108.8 (2)	H27C—C27B—H27H	123.8
C19—N1—C22	125.0 (3)	H27D—C27B—H27H	106.3
C20—N1—C22	126.2 (2)	O1B—C27B—H27I	106.3
N1—C19—N2	108.9 (2)	C28B—C27B—H27I	112.2
N1—C19—H19A	125.6	H27C—C27B—H27I	8.5
N2—C19—H19A	125.6	H27D—C27B—H27I	114.5
C19—N2—C21	108.1 (2)	H27H—C27B—H27I	116.1
C19—N2—C23	125.3 (2)	O1B—C27B—H27J	102.2
C21—N2—C23	126.5 (2)	C28B—C27B—H27J	107.0
C21—C20—N1	106.7 (2)	H27C—C27B—H27J	111.0
C21—C20—H20A	126.6	H27D—C27B—H27J	8.0
N1—C20—H20A	126.6	H27H—C27B—H27J	102.2
C20—C21—N2	107.5 (2)	H27I—C27B—H27J	111.6
C20—C21—H21A	126.3	C27B—C28B—H28C	112.3
N2—C21—H21A	126.3	C27B—C28B—H28D	107.8
N1—C22—H22A	109.5	H28C—C28B—H28D	107.5
N1—C22—H22B	109.5		

### Hydrogen-bond geometry (Å, º)

**Table:**

*D*—H···*A*	*D*—H	H···*A*	*D*···*A*	*D*—H···*A*
O1*B*—H1*BA*···N1	0.85	2.68	3.36 (1)	139
C19—H19*A*···O1*A*	0.95	2.59	3.17 (1)	120
C2—H2*A*···Br2	0.95	2.95	3.817 (2)	153
C23—H23*A*···Br3^i^	0.99	2.84	3.718 (3)	148
C20—H20*A*···Br1^ii^	0.95	3.02	3.906 (3)	156
C8—H8*A*···Br1	0.95	3.08	3.921 (2)	149
C23—H23*B*···Br2^iii^	0.99	3.13	3.711 (3)	119

Symmetry codes: (i) −*x*+1, −*y*+1, −*z*+1; (ii) *x*+1/2, −*y*+1/2, *z*+1/2; (iii) *x*−1/2, −*y*+1/2, *z*+1/2.
